# Investigating a clinically actionable *BRAF* mutation for monitoring low-grade serous ovarian cancer: A case report

**DOI:** 10.1016/j.crwh.2022.e00395

**Published:** 2022-02-06

**Authors:** R. Silva, B. Moran, S. Das, N. Mulligan, M. Doughty, A. Treacy, K. Sheahan, C.M. Kelly, A.G. Duffy, A.S. Perry, D.J. Brennan

**Affiliations:** a Cancer Biology and Therapeutics Laboratory, UCD Conway Institute of Biomolecular and Biomedical Research, University College Dublin, Dublin, Ireland; b Systems Biology Ireland, UCD School of Medicine, University College Dublin, Dublin, Ireland; c School of Biology and Environmental Science, University College Dublin, Dublin, Ireland; d Department of Pathology, St Vincent's University Hospital, Dublin, Ireland; e School of Pharmacy and Biomolecular Sciences, Royal College of Surgeons in Ireland, Dublin, Ireland; f Department of Pathology, Mater Misericordiae University Hospital, Dublin, Ireland; g Department of Oncology, Mater Misericordiae University Hospital, Dublin, Ireland; h UCD Gynaecological Oncology Group, UCD School of Medicine, Mater Misericordiae University Hospital, Dublin, Ireland; i UCD School of Medicine, National Maternity Hospital, Dublin, Ireland

**Keywords:** Low-grade serous ovarian cancer, Cell-free circulating DNA, Targeted therapy, Next-generation sequencing, Digital droplet PCR

## Abstract

Low-grade serous ovarian cancer (LGSOC) poses a specific clinical challenge due to advanced presentation at diagnosis and the lack of effective systemic treatments. The aim of this study was to use a precision medicine approach to identify clinically actionable mutations in a patient with recurrent LGSOC. Primary, metastatic and recurrence tissue, and blood samples were collected from a stage IV LGSOC patient. Single-gene testing for clinically actionable mutations (*BRAF* V600, *KRAS* and *NRAS*) and subsequent whole-exome sequencing (WES) were performed. Droplet digital PCR was used to evaluate the presence of an identified *BRAF* D594G mutation in the matched plasma cell-free DNA (cfDNA). No clinically actionable mutations were identified using single-gene testing. WES identified a *BRAF* D594G mutation in six of seven tumor samples. The patient was commenced on a MEK inhibitor, trametinib, but with minimal clinical response. A newly designed ddPCR assay detected the *BRAF* alteration in the matched tissues and liquid biopsy cfDNA. The identification and sensitive plasma detection of a common “druggable” target emphasises the impact of precision medicine on the management of rare tumors and its potential contribution to novel monitoring regimens in this field.

## Highlights

• First report of *BRAF* D594G mutation in multiple samples of a recurrent LGSOC patient.
• Discovery of a *BRAF* actionable mutation expands the range of LGSOC therapeutic options.
• ddPCR assay allows sensitive detection of the mutation in tissue and plasma samples.

## 1 Introduction

Ovarian cancer (OC) is the most lethal gynaecological malignancy, accounting for over 180,000 yearly cancer deaths worldwide [1]. Serous OC is the most common histological subtype of OC, and while high-grade serous ovarian cancer (HGSOC) accounts for most serous epithelial OCs (~95%), a small number of women present with low-grade serous ovarian cancer (LGSOC) [2]. Most women present with advanced disease at diagnosis, that is generally resistant to systemic chemotherapy and anti-endocrine therapy, and eventually succumb to their disease [3]. There is thus a significant unmet need for more effective therapeutic approaches for LGSOC.

To improve the treatment modalities currently used in LGSOC, research has focused on understanding the molecular landscape of this rare cancer type. LGSOC is typically characterised by a high frequency of activating mutations in upstream regulators of the mitogen-activated protein (MAPK) pathway, such as *KRAS, NRAS* and *BRAF* [4,5]. This suggests that the use of BRAF or MEK inhibitors may have some efficacy in LGSOC [6].

In addition to the observed chemoresistance and dearth of effective therapeutic options, LGSOCs also lack an appropriate follow-up regimen, as the use of standard clinical prognostic and predictive tools used in the context of OC have shown limited utility in LGSOC [7]. Liquid biopsies offer a minimally invasive and sensitive technique to complement existing monitoring tools, via cell-free circulating tumor DNA (ctDNA) or circulating tumor cells (CTCs) [8].

In this case report, we demonstrate how the identification of a clinically actionable genomic alteration led to the development of a novel liquid biopsy assay that could potentially enable surveillance of LGSOC patients on targeted therapy.

## 2 Case Presentation

**Fig. 1:**
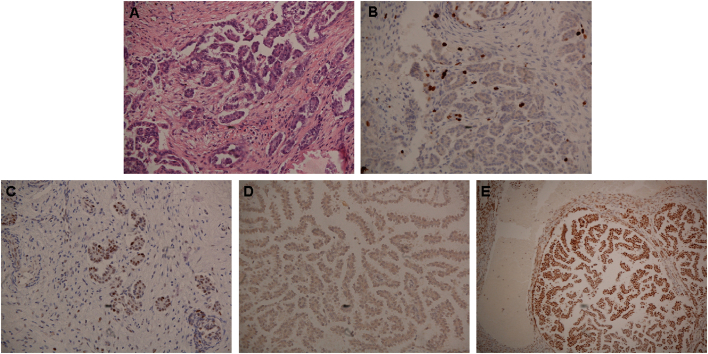
Immunohistochemical (IHC) staining of tumor sections. **A)** Hematoxylin and eosin staining of patient's tumor section showing histological findings that are compatible with low-grade serous ovarian cancer. **B)** Low immunoexpression of Ki-67 protein, which is associated with low proliferative tumors. Wild type like and negative expression patterns were observed for **C)** p53 and **D)** BRAF V600E proteins, respectively. **E)** Microsatellite instability testing demonstrated high expression of mismatch repair proteins, indicative of a MSI stable tumor. IHC of MLH1 protein is shown as an example.

**Fig. 2:**
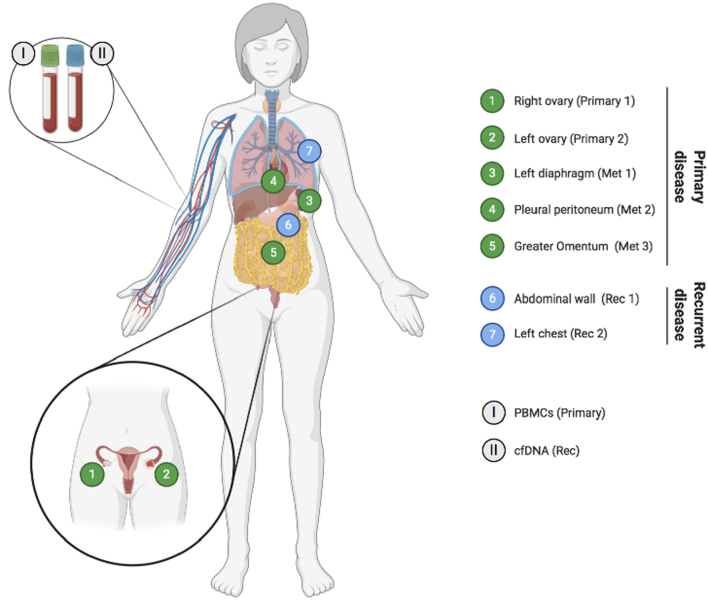
Overview of analysed specimens. Anatomical location of samples collected during either primary cytoreductive surgery (green) or second surgery (upon recurrence; blue). Whole blood was also collected during both surgeries. Abbreviations: cfDNA – cell-free DNA; Met – metastasis; PBMC – peripheral blood mononuclear cell; Rec – recurrence. (For interpretation of the references to colour in this figure legend, the reader is referred to the web version of this article.)

A 43-year-old woman presented with large-volume ascites, bilateral pleural effusions and pelvic masses, and a large-volume omental disease. A laparoscopy showed widespread peritoneal carcinomatosis, and a biopsy confirmed the diagnosis of LGSOC. Immunohistochemistry showed a p53 wild type pattern, with low proliferation index, expression of oestrogen and progesterone receptors, microsatellite stable tumor (Fig. 1A-E**)**. She proceeded to have primary cytoreductive surgery, which included a complete abdomeno-pelvic peritonectomy, greater and lesser omentectomy, splenectomy, cholecystectomy, total colectomy and abdominal hysterectomy, and bilateral salpingo-oophorectomy. Residual disease was 0.5 cm, located on the small bowel mesentery. The completeness of cytoreduction score (CC score) was 2. Tissues from the right and left ovaries (primary tumor) and three other metastatic sites (greater omentum, left diaphragm and pleural peritoneum) were acquired (Fig. 2) and post-operative histology confirmed LGSOC in all specimens. The patient subsequently completed six cycles of adjuvant carboplatinum and paclitaxel and was started on maintenance tamoxifen.

Eleven months later, she presented with increasing shortness of breath. A CT scan of the thorax/abdomen/pelvis demonstrated a new left-sided pleural effusion and an abdominal wall mass, which biopsy confirmed as recurrent LGSOC. She underwent a left-sided video-assisted thoracoscopic surgery (VATS) procedure and talc pleurodesis, and tissues from two other sites (abdominal wall and left chest) were collected (Fig. 2). A decision was made to change her anti-endocrine therapy from tamoxifen to letrozole.

**Fig. 3:**
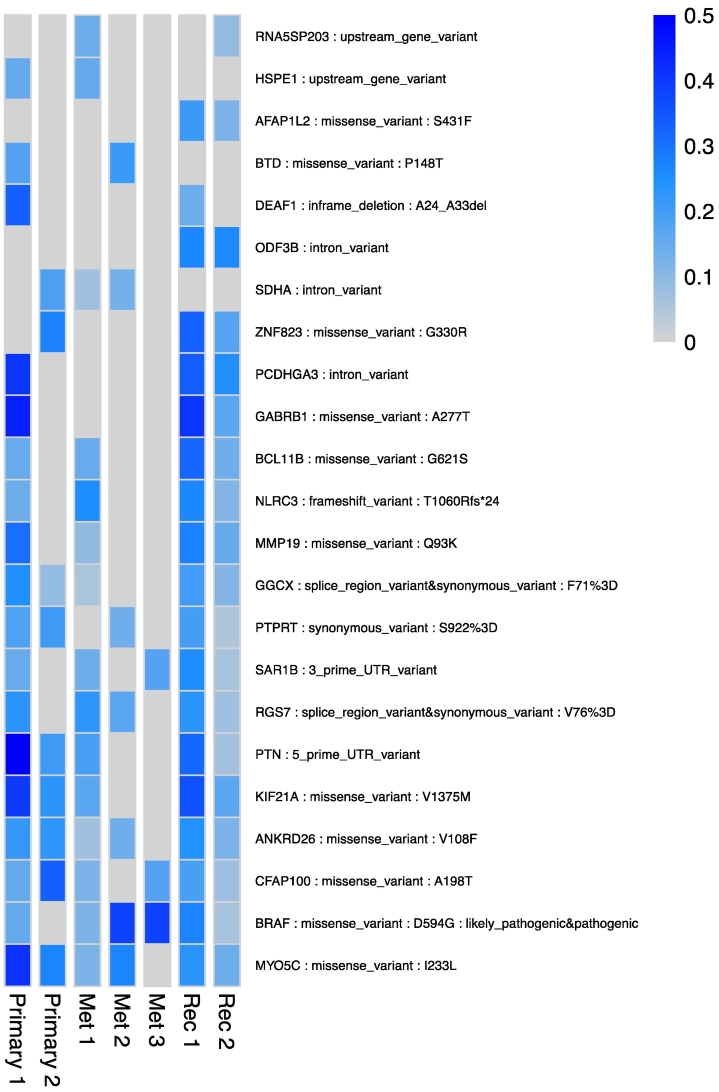
SNVs detected in primary and recurrent LGSOC samples. Representation of the 23 somatic single nucleotide variants (SNVs) detected. Differences in mutant allele fraction (MAF) are represented by the blue colour gradient, blue: present, grey: absent. (For interpretation of the references to colour in this figure legend, the reader is referred to the web version of this article.)

To investigate possible causes for the observed therapy resistance and identify new therapeutic options, we employed a multistep genomic approach to determine the molecular characteristics of this patient's tumor (**Supplementary Material**). While single-gene testing revealed no alterations for *EGFR*, *NRAS*, *KRAS* and *BRAF* in all analysed tissue samples (*n* = 7; 2 primary, 3 metastatic and 2 recurrent sites), whole-exome sequencing (WES) identified 23 protein-coding somatic SNVs across all studied samples (Fig. 3). The majority (90%) were classified as missense variants, including a clinically relevant *BRAF* mutation (D594G), which has previously been linked to several cancers (i.e., melanoma, colorectal and lung) [9,10], but has been previously described only once in a patient-derived LGSOC cell line [11,12]. Unfortunately, the disease continued to progress while the patient was on letrozole and, given the minimal therapeutic options available and the known presence of a *BRAF* mutation, a decision was made that this patient should commence the MEK1/2 inhibitor trametinib. However, she showed minimal clinical response and succumbed to her disease two months later.

**Fig. 4:**
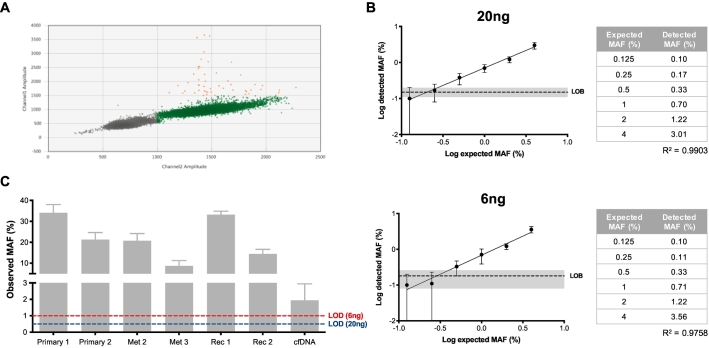
Detection of *BRAF* D594G mutation by ddPCR. **A)** Limit of blank (LOB) determination for 20 ng DNA samples. 16-replicate wells of wild type-only template were run. Droplets are classified as wild type-only (green), mutant-only droplets (blue) double wild type-mutant droplets (orange) and double negative (grey). The LOB was set at 0.15% and 0.18% for 20 and 6 ng samples, respectively. **B)** Limit of detection (LOD) determination for DNA amounts of 20 (upper) and 6 ng (lower). Log of MAF values was used to calculate correlation between expected and detected MAFs (R^2^). Confidence interval for LOB is shown in grey. Based on obtained values (table), LOD was determined as 0.5% and 1% for 20 and 6 ng, respectively. **C)** MAF observed for tested tissue and cfDNA samples. Abbreviations: cfDNA – cell-free DNA; MAF – mutant allele fraction. (For interpretation of the references to colour in this figure legend, the reader is referred to the web version of this article.)

Having identified a novel *BRAF* mutation in tumor samples, we sought to develop a sensitive droplet digital PCR (ddPCR) assay to detect the *BRAF* D5945G mutation in plasma cfDNA (**Supplementary Material**). Evaluation of the sensitivity of the assay was performed with 20 ng (tissue sample input) and 6 ng (cfDNA sample input) and a limit of detection was established (Fig. 4A-B). We were able to detect the *BRAF* mutation in all tested tissue samples (*n* = 6; left diaphragm site – Met 1 – was not available) (Fig. 4C). Interestingly, one of the primary tumor samples (left ovary – primary 2), previously revealed to be negative by WES analysis, was positive for the presence of the mutation when assayed with ddPCR. Additionally, we also detected the mutation in a cfDNA sample collected before the patient commenced trametinib (Fig. 4C).

## 3 Discussion

Using a stepwise genomic approach, we identified a *BRAF* D594G mutation in multiple tissue samples from the same patient, suggesting that altered MAPK signalling was an important aspect of this particular tumor. *BRAF* mutations are present in up to 14% of LGSOC, with the majority of those corresponding to *BRAF* V600E alterations [13]. The D594G mutation, designated as a class 3 *BRAF* mutant, causes inactivation of this protein and subsequent impaired kinase activity [9]. Due to this inactivation, tumors harbouring this mutation are unlikely to respond to established *BRAF* inhibitors, which generally selectively target a conformational change only present in *BRAF* V600-mutant activated proteins. Although unable to directly phosphorylate MEK, these “kinase-dead” mutants still exhibit some signalling activity in the MAPK pathway. Heidorn et al. demonstrated that class 3 *BRAF* mutants have the ability to bind and activate CRAF, in a RAS-dependent manner, leading to CRAF hyperactivation and subsequent elevated MEK and ERK signalling [14]. Prior to this discovery, this mutation had been reported by only one other group, in a patient-derived LGSOC cell line [11,12], which highlights its rarity in this disease. In fact, the authors further report that of all the mutations observed in their established LGSOC cell lines, only D594G and another *NRAS* mutation were not found in the GENIE cohort, which includes over 97 LGSOC tumor specimens [12,15]. Although MEK inhibitor sensitivity *in vitro* was also tested in this study, this report gives an insight into the use of MEK inhibitors *in vivo*, and provides useful information that can be translated to the clinic, and could otherwise be biased by the use of a cell line (i.e. lack of tumor microenvironment or drug-cell interactions). Additionally, this is the first study that identified this particular *BRAF* variant in plasma cell-free DNA, highlighting the potential of using liquid biopsies as a minimally invasive alternative for identification of new actionable targets, and subsequent therapeutic monitoring in LGSOC, as previously observed for other malignancies [16,17].

This data, combined with increasingly large cohorts demonstrating the prevalence of genomic alterations in *BRAF*, *NRAS* and *KRAS* in LGSOC [18,19], suggests that a variety of tyrosine kinase inhibitors warrant further investigation in LGSOC. A number of clinical trials have sought to exploit this therapeutic approach in recurrent LGSOC, with a particular focus in MEK inhibitors. An open-label, single-arm phase II trial of the MEK inhibitor selumetinib showed a 15% response rate and a median progression-free survival time of 11 months. Despite mutational data being available for the majority of the cohort, no correlation was found between any *BRAF* (codon 599 only) or *KRAS* (codons 12/13) mutation and therapeutic response [20]. Two other phase II/III trials compared two MEK inhibitors against physician's choice of chemotherapy/hormonal therapy in women with persistent or recurrent LGSOC. The MILO/ENGOT-ov11 study failed to demonstrate an improvement in progression-free survival for binimetinib; however, a post-hoc analysis suggested that responses rates were higher in *KRAS* mutated compared with *KRAS* wild type LGSOC (objective response rate 44% vs 19%) [21]. A second randomised phase II/III trial compared trametinib to physician's choice, and demonstrated a significant improvement in objective response rates (26% v 6%) and improved progression-free survival (13 vs 7.2 months) [22]. Based on these findings, we decided to administer trametinib to the patient highlighted in this report.

The advent of liquid biopsies has contributed towards the potential integration of personalised cancer medicine in the clinical management of OC patients. In fact, the use of liquid biopsy approaches has been associated with superior levels of prediction of therapeutic outcome in the OC setting. For example, Parkinson et al. reported that a reduction of ≤60% in *TP53* MAF in plasma after one cycle of cytotoxic platinum-based chemotherapy was associated with a shorter time to progression [23]. Although the vast majority of liquid biopsy studies have been conducted in HGSOC patients, these reports highlight the reliability of using ctDNA for prediction of recurrence and treatment response. We were able to use a novel ddPCR assay to detect the presence of the *BRAF* D594G mutation in both tissue and plasma samples from a patient with LGSOC. Notably, one of the tissue samples that was determined as negative by WES (primary 2) was positive when evaluated by ddPCR. Compared with ddPCR, WES has lower detection sensitivity (~0.001% for ddPCR and ~ 5–10% for WES). Although the ddPCR-determined MAF for this sample was above the usual WES LOD, we believe that this was a false-negative result which stemmed from low sequencing coverage. Compared with the MAF obtained with the tissue samples, detection of the mutation in plasma cfDNA was lower, especially for a recurrent patient. This observation might be a result of cfDNA containing DNA from multiple cell sources, of which tumor is only a portion, as opposed to samples derived from the tumor itself. Nevertheless, detection was still above the determined limit for this assay and these results are on par with other reports evaluating the presence of alterations in cfDNA [24]. Thus, these results provide support for further evaluation of liquid biopsy-based therapeutic response monitoring in OC.

Technological advances have improved our understanding of the underlying molecular features of rare tumors such as LGSOC and, as a result, the clinical management of LGSOC has been slowly evolving from a “one-size-fits-all” regimen to a more personalised approach. Here, the clinical application of WES and ddPCR as useful tools for managing a recurrent LGSOC case are demonstrated. Further analysis, in a series of patients, is required to understand how to fully implement these personalised approaches into the clinical management of OC.

## Appendix A Supplementary data

Supplementary material Image 1
