# Supplementary material for: Investigating a clinically actionable BRAF mutation for monitoring low-grade serous ovarian cancer: A case report
Source: Case Rep Womens Health. 2022 Feb 6;34:e00395. doi: 10.1016/j.crwh.2022.e00395 (PMC8851090; doi:10.1016/j.crwh.2022.e00395)
Supplement: Supplementary file 1 — Supplementary material [file mmc1.docx]

**Development of a novel, clinically actionable ddPCR assay for monitoring low-grade serous ovarian cancer: a case report**

**Supplemental Material**

**METHODS**

**Clinical specimens**

Informed consent was obtained from a LGSOC patient for the use of collected clinical specimens and publication of this case report.

A total of 9 samples were collected at Mater Misericordiae University Hospital, Dublin, during the clinical course of the disease (**Fig. 1 – main manuscript**). Tissues from the right and left ovaries (primary tumour) and three other metastatic sites (greater omentum, left diaphragm and pleural peritoneum) were acquired during primary cytoreductive surgery (November 2016). Tissues from two other sites (abdominal wall and left chest – cells obtained from needle biopsy) were collected during a second surgery upon disease recurrence (October 2017). All tissue samples were preserved in formalin-fixed paraffin embedded (FFPE) blocks; each containing >50% tumour (range: 50-80%). 20 mL of whole blood was collected at the first cytoreductive surgery for isolation of peripheral blood mononuclear cells (PBMCs) and at disease recurrence, for isolation of cell-free DNA (cfDNA).

**DNA isolation**

For each tissue sample, five 4uM sections from the FFPE blocks were subjected to deparaffinization, and macrodissection to obtain tumour material. DNA was isolated from scraped material using the QIAamp DNA FFPE tissue (Qiagen). DNA from PBMCs and plasma was extracted using DNeasy Blood & Tissue and QIAamp Circulating Nucleic Acid (Qiagen), respectively. Isolated DNA samples were quantified by the Qubit High-Sensitivity DNA quantification kit (Invitrogen) and stored at -20⁰C until further use. Total DNA concentration for cfDNA (0.754 ng/μL) was less than that obtained for the tissue samples (average: 22.27 ng/μL; range: 5.06-58 ng/μL) and PBMCs (49.2 ng/μL).

**Immunohistochemistry (IHC) and microsatellite instability (MSI) testing**

IHC was performed for Ki-67, p53 and BRAF V600E using anti-human Ki-67 MIB-1 clone (Dako), anti-p53 DO7 clone (Leica) and anti-BRAF V600E VE1 clone (Ventana) antibodies on the Ventana Benchmark Ultra platform slide staining system (Arizona, USA). The process involved cell conditioning for 64 minutes (with a Tris-based buffer), pre-oxidation inhibition and primary antibody incubation for 16 minutes at 36ºC. Ventana optiview DAB IHC detection kit was used to detect protein expression. The slides were counterstained with Ventana hematoxylin and bluing reagent for 4 minutes.

Mismatch repair (MMR) status was assessed using IHC for MMR proteins (MMRPs): hMLH1 (BD Bioscience, clone G168-728), hPMS2 (Bd Biosciences, clone A16-4), hMSH2 (Calbiochem, clone FE11) and hMSH6 (BD Biosciences, clone 44). Automated IHC was performed on the BOND instrument (Leica). The protocol involves heat-induced antigen retrieval with BOND Epitope Retrieval 2 solution for 30 minutes. Slides were incubated with each respective antibody (1:200 dilution) for 15 minutes at room temperature. Visualisation of the antibody antigen reaction was via the BOND polymer Refine Detection kit. Nuclear staining in any area of the tumour was classified as showing no loss of the MMRPs.

**Single gene testing**

Actionable EGFR, BRAF and NRAS mutations were screened for with the Cobas BRAF/NRAS Mutation Test LSR kit (Roche), performed according to manufacturer’s instructions.

**Whole-exome sequencing and data processing**

Whole-exome sequencing (WES) data were generated using 50ng DNA extracted from tissue samples from 7 different sites and PBMCs. Genomic libraries were prepared using Nextera® Rapid Capture Exomes kit (Illumina) and quantified with the Kapa Library Quantification kit (Roche), according to manufacturer’s protocol. The NextSeq 500 platform (Illumina) was used to sequence contents of the libraries in a 2x75bp run.

Sequencing data were processed using the ‘somatic_n-of-1’ workflow composed in Nextflow [16] ([github.com/brucemoran/somatic_n-of-1](http://github.com/brucemoran/somatic_n-of-1) commit:<COMMIT>). A Singularity container [17, 18] was used for the environment required, which was built largely using the *conda* package (doc.conda.io). The workflow contains processes for: quality control of sequencing data, using ‘fastp’ and ‘fastqc’ [19, 20]; trimming of adapters and low quality bases using ‘BBDuk’ [21]; alignment using ‘BWA MEM’ [22]; best-practices ‘Genome Analysis ToolKit’ (GATK) processing [23]; germline mutation calling using ‘HaplotypeCaller’ [24]; multiple metrics of aligned and GATK-processed data using a suite of Picard tools ([broadinstitute.github.io/picard/](http://broadinstitute.github.io/picard/)); copy number analysis using ‘Facets’ [25]; somatic variant calling using ‘MuTect2’ [26](SNV and indels), ‘Manta’ [27] and ‘Strelka2’ [28] (indels and SNV, respectively) and ‘Lancet’ [29](SNV); variant annotation using the ‘Variant Effect Predictor’ (VEP) [30]; ensemble variant calling using the ‘somenone’ R package ([github.com/brucemoran/somenone](https://github.com/brucemoran)); reporting using the ‘Personalised Cancer Genome Reporter’ (PCGR) [31] for somatic variants, and the ‘Cancer Predisposition Sequencing Reporter’ (CPSR; [github.com/sigven/cpsr](https://github.com/sigven/cpsr)) for germline variants; and ‘MultiQC’ [32] to compile all quality control and metrics from processes into a single HTML document.

**Droplet digital PCR (ddPCR) workflow**

A total of six tissue samples (one of the samples, Met 1, did not have sufficient DNA for analysis) and one cfDNA were analysed for the presence of the *BRAF* D594G mutation found through WES analysis. Mutant and wild type *BRAF* sequences were used as a template for designing ddPCR assays.

Custom PrimeTime® assays composed of primers and double-quenched probes, containing either a 5’-FAM™ (mutant) or 5’-HEX™ (wild type) reporter dye, were ordered from Integrated DNA Technologies Inc (IDT). Wild type and mutant *BRAF* gBlock synthetic DNA fragments were also ordered (**Supplemental Table 1**).

Each 20μl ddPCR reaction contained 10μl of 2x ddPCR Super Mix for probes (Bio-Rad), 1μl of wild type PrimeTime® assay, 1μl of mutant PrimeTime® assay and up to 8μl of DNA/ddH_2_0. Following droplet generation on the QX200™ droplet generator (Bio-Rad), the plate was sealed and PCR was performed on a T100™ thermal cycler (Bio-Rad). Optimization of the ddPCR annealing temperature was carried out by doing a temperature gradient PCR of 55º-65ºC, and the optimal temperature was established at 58.8ºC. Final thermal cycling conditions were as follow: 10min activation step at 95ºC, followed by 40 cycles of denaturation at 95ºC (15s) and annealing/extension at 58.8ºC (90s), and a 10 min deactivation step at 98ºC. After a 10min incubation at room temperature, the plate was read using a QX200™ droplet reader (Bio-Rad).

Evaluation of the limit of blank (LOB) and the limit of detection (LOD) were carried out to test assay performance. For LOB, 16-replicate wild type-only (mutation-negative) controls were run to identify any false-positives. For LOD, a serial dilution of mutant template, in a constant wild type background, was used to determine the lowest mutant concentration that can be distinguished from the LOB. Each dilution was run in quadruplicate and the expected mutant allelic fraction (MAF) of these dilutions ranged from 0.125-4%. No template controls (NTC) were run in every experiment.

QuantaSoft™ Analysis Pro software (Bio-Rad, v.1.0.596) was for analysis. Thresholds for positivity were set manually and used to calculate predicted MAF. All wells with <10,000 droplets were excluded from further analysis.

**Statistical analysis**

After adjusting thresholds and counting the fraction of positive droplets, estimation of target concentration and MAF for each sample was obtained using binomial Poisson statistics (with 95% confidence intervals) [33]. Linear regression analysis was used to determine the relationship between observed and expected MAFs after a ddPCR run.

**TABLES:**

**Supplemental Table 1. Primers, probes and gBlock synthetic DNA fragment for ddPCR**

| **Gene** | **Description** | **Sequence** | **Amplicon size (bp)** |
| --- | --- | --- | --- |
| **BRAF**  **(D594G)** | **Primer (Forward)** | CCTTTACTTACTACACCTCAG | **95** |
|  | **Primer (Reverse)** | ACTCCATCGAGATTTCACT |  |
|  | **Probe wild type** | TAGGTG**A**TTTTGGTCTAGCT |  |
|  | **Probe mutant** | TAGGTG**G**TTTTGGTCTAGC |  |
|  | **Gblock wild type** | ATTAGATCTCTTACCTAAACTCTTCATAATGCTTGCTCTGATAGGAAAATGAGATCTACTGTTTTCCTTTACTTACTACACCTCAGATATATTTCTTCATGAAGACCTCACAGTAAAAATAGGTG**A**TTTTGGTCTAGCTACAGTGAAATCTCGATGGAGTGGGTCCCATCAGTTTGAACAGTTGTCTGGATCCATTTTGTGGATGGTAAGAATTGAGGCTATTTTTCCACTGATTAAATTTTTGGCCCTGA | **251** |
|  | **Gblock mutant** | ATTAGATCTCTTACCTAAACTCTTCATAATGCTTGCTCTGATAGGAAAATGAGATCTACTGTTTTCCTTTACTTACTACACCTCAGATATATTTCTTCATGAAGACCTCACAGTAAAAATAGGTG**G**TTTTGGTCTAGCTACAGTGAAATCTCGATGGAGTGGGTCCCATCAGTTTGAACAGTTGTCTGGATCCATTTTGTGGATGGTAAGAATTGAGGCTATTTTTCCACTGATTAAATTTTTGGCCCTGA |  |
